# Supplementary material for: Tuning of the flat band and its impact on superconductivity in Mo5Si3−xPx
Source: Nat Commun. 2024 Mar 11;15:2197. doi: 10.1038/s41467-024-46514-2 (PMC10928102; doi:10.1038/s41467-024-46514-2)
Supplement: Supplementary file 1 — Supplementary Information [file 41467_2024_46514_MOESM1_ESM.pdf]

Supplemental Materials for "Tuning of the flat band and its impact on superconductivity in $Mo_5Si_{3-x}P_x$"

Rustem Khasanov,[1,*] Bin-Bin Ruan,[2,†] Yun-Qing Shi,[2,3] Gen-Fu Chen,[2,3] Hubertus Luetkens,[1] Zhi-An Ren,[2,3] and Zurab Guguchia[1]

[1]*Laboratory for Muon Spin Spectroscopy, Paul Scherrer Institute, CH-5232 Villigen PSI, Switzerland*
[2]*Institute of Physics and Beijing National Laboratory for Condensed Matter Physics, Chinese Academy of Sciences, Beijing 100190, China*
[3]*School of Physical Sciences, University of Chinese Academy of Sciences, Beijing 100049, China*

I. X-RAY DIFFRACTION DATA

XRD patterns of polycrystalline $Mo_5Si_{3-x}P_x$ ($0 \leq x \leq 1.6$) are presented in Figure 1 (a). The main peaks were identified with the W_5Si_3 type structure (space group $I4/mcm$). The peaks marked by asterisks correspond to the impurity Mo_3P fraction.

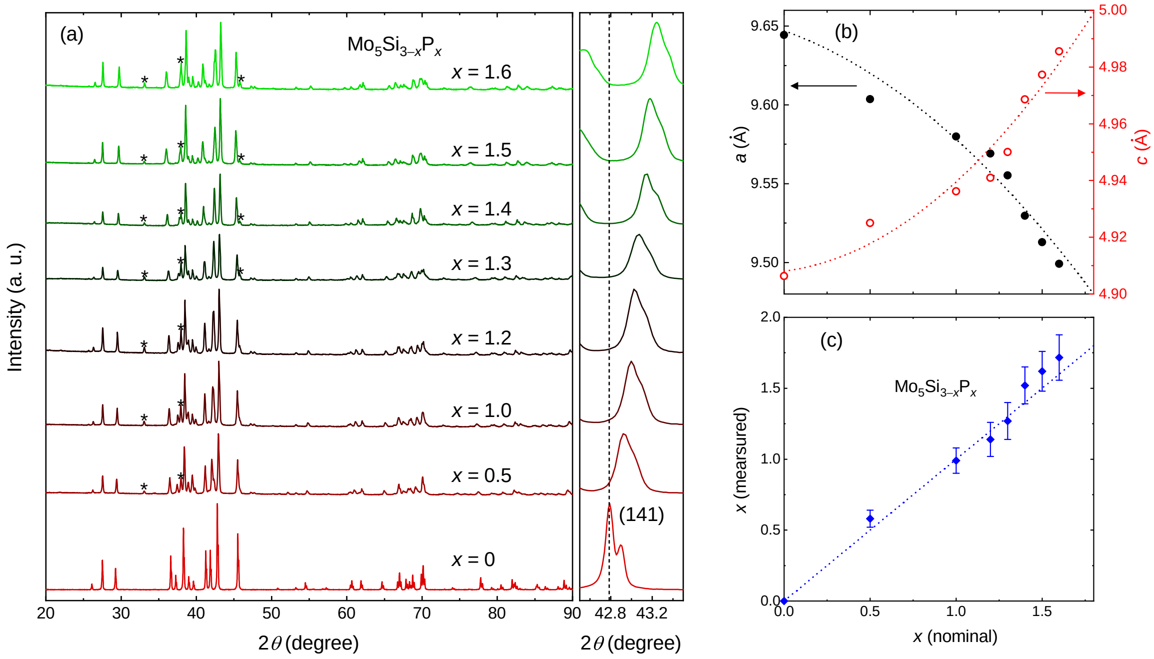

FIG. 1: (a) Powder x-ray diffraction patterns of $Mo_5Si_{3-x}P_x$ ($0 \leq x \leq 1.6$). The peaks of the Mo_3P impurity phase are marked by asterisks. The inset shows the position of (141) peak of $Mo_5Si_{3-x}P_x$ at various doping levels. (b) The dependence of the lattice parameters a and c on the nominal phosphorus content x. (c) The measured phosphorus content versus its nominal value. The dashed line represents the case where the measured values are equal to the nominal ones.

A successful phosphorus doping into the Mo_5Si_3 matrix is confirmed by the corresponding changes of $a-$ and $c-$lattice constants, as shown in Fig. 1 (b). The phosphorus content in synthesized $Mo_5Si_{3-x}P_x$ samples was determined by comparing the lattice parameters a and c with those reported in Ref. 1. Figure 1 (c) implies that the measured P concentrations agree well with the nominal ones. Consequently, the nominal phosphorus content x was used for representing the data in the main text.

II. RESISTIVITY DATA

Figure 2 shows the resistivity curves normalized to the values at $T = 16$ K $[R(T)/R(16\text{ K})]$ measured in magnetic fields ranging from 0.0 to 9.0 T. The superconducting transition temperature at the applied field $\mu_0 H_{\rm ap}$ $[T_{\rm c}(\mu_0 H_{\rm ap})]$ was determined as a cross point of $R(T, H_{\rm ap})(T)$ curve with $R(T)/R(16\text{ K}) = 0.5$ line, see the top left panel of Fig. 2.

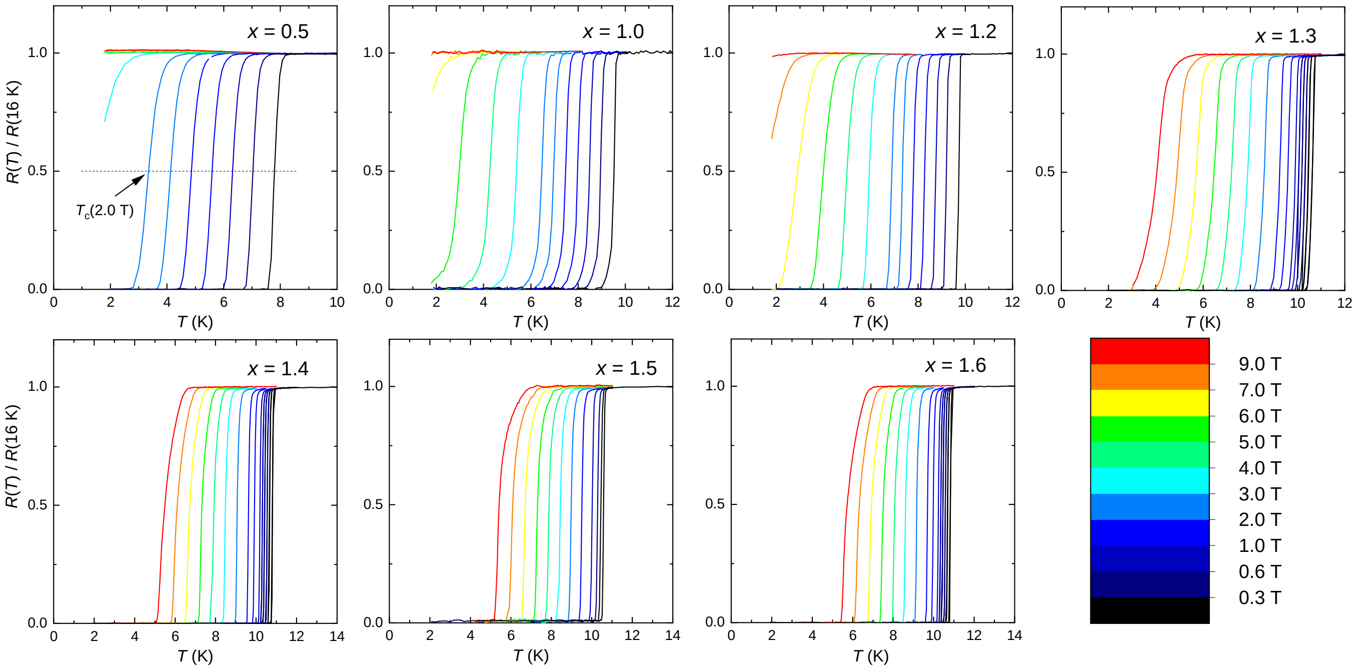

FIG. 2: Temperature dependencies of resistivity of $Mo_5Si_{3-x}P_x$ under magnetic fields ranging from 0.0 to 9.0 T. The superconducting transition temperature T_c is defined from the midpoint of $R(T, H_{ap})$ curves [*i.e.*, as the value where $R(T)/R(16\text{ K}) = 0.5$, see the top left panel].

III. SPECIFIC HEAT DATA

The temperature dependencies of the specific heat (C_p) of $Mo_5Si_{3-x}P_x$ are presented in Fig. 3 (a). Note that the $C_p(T)$ data were corrected by subtracting the minor contributions of Mo_3P.[1]

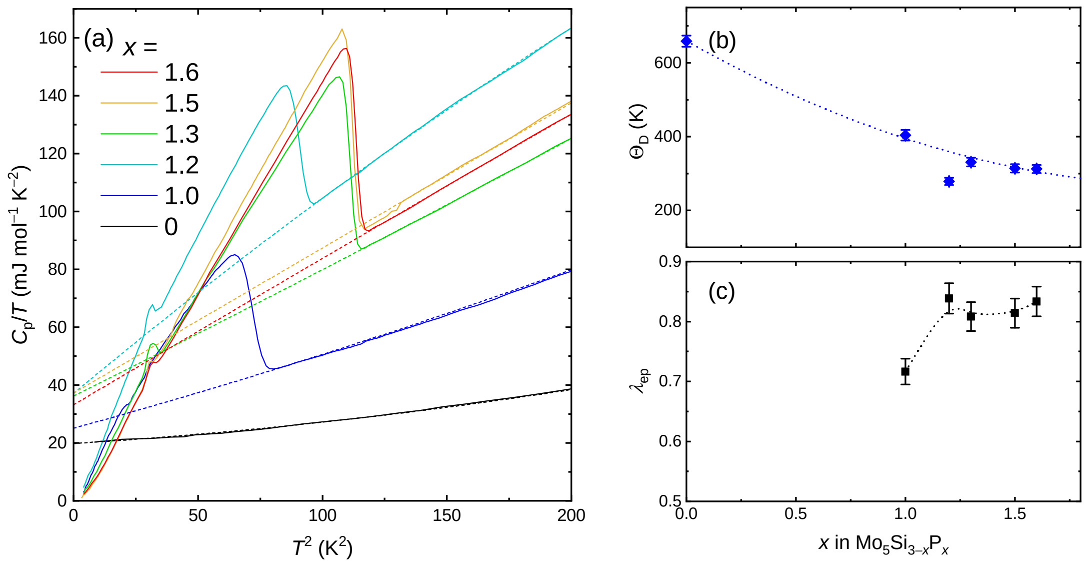

FIG. 3: (a) Temperature dependencies of the specific heat C_p of $Mo_5Si_{3-x}P_x$ under zero magnetic field. The dash lines correspond to the fit of the Debye model (Eq. 1) to the $C_p(T)$ data. The kinks at $T^2 \simeq 30$ K arise from the Mo_3P impurity. (b) Dependence of the Debye temperature (Θ_D) on the phosphorus content x. (c) Dependence of the electron-phonon coupling constant (λ_{ep}) on x.

The temperature evolution of C_p in the normal state (*i.e.*, for $T_c < T \lesssim 16$ K) was analyzed within the framework

of the Debye model:

$$C_{\rm p}(T) = \gamma T + \beta T^3 + \delta T^5, \tag{1}$$

where the linear T term represents the electronic specific heat contribution γ, while T^3 and T^5 terms account for the harmonic and anharmonic phonon contributions, respectively. Fits of the Debye model to $C_{\rm p}(T)$ data are presented in Fig. 3 (a) by dotted lines.

The Debye temperature $\Theta_{\rm D}$ was further obtained by using the 'harmonic' term β as:

$$\Theta_{\rm D} = (12\pi^4 NR/5\beta)^{1/3}. \tag{2}$$

Here N is the number of atoms per formula unit, and R is the ideal gas constant. The dependence of $\Theta_{\rm D}$ on x is shown in Fig. 3 (b).

The electron-phonon coupling constant $\lambda_{\rm ep}$ [Fig. 3 (c)] was estimated from the McMillan equation:[2]

$$\lambda_{ep} = \frac{1.04 + \mu^* \ln(\Theta_D/1.45T_c)}{(1 - 0.62\mu^*)\ln(\Theta_D/1.45T_c) - 1.04} \tag{3}$$

by using the Coulomb screening parameter $\mu^* = 0.13$.[1]

IV. DOPING DEPENDENCE OF THE DENSITY OF STATES

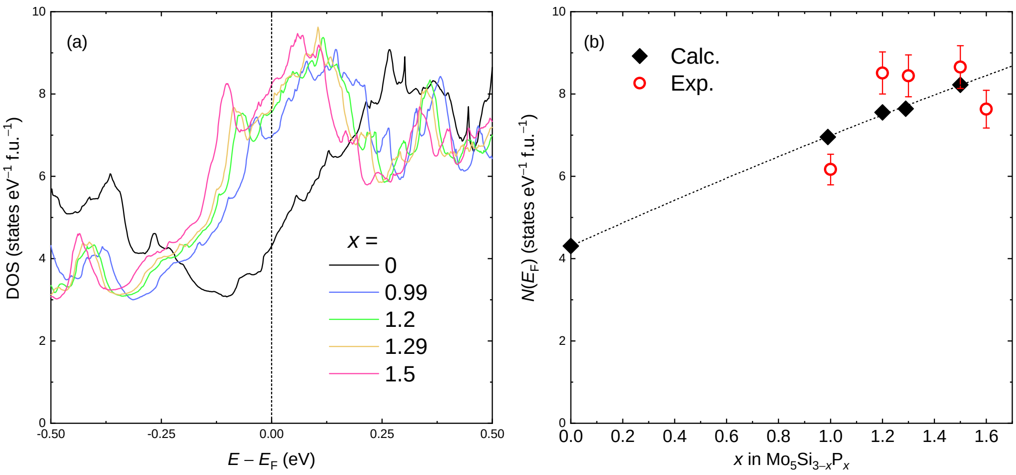

FIG. 4: (a) Calculated density of states (DOS) of $Mo_5Si_{3-x}P_x$ near the Fermi level ($E_{\rm F}$). (b) Comparison of the calculated and experimental DOS on $E_{\rm F}$ [$N(E_{\rm F})$] of $Mo_5Si_{3-x}P_x$.

The absence of a competing ordered state(s), which might be responsible for reducing the number of carriers accessible for the superconducting condensate, was checked by comparing the Density of States (DOS) as obtained from the first principle calculations [Fig. 4 (a)] with DOS at the Fermi level [$N(E_{\rm F})$] determined from the above reported quantities (γ and $\lambda_{\rm ep}$) via:

$$\gamma = \frac{1}{3} N(E_{\rm F})\pi^2 k_B^2 (1 + \lambda_{\rm ep}). \tag{4}$$

An agreement between the 'theoretical' and 'experimental' dependencies of $N(E_{\rm F})$ on x, as presented in Fig. 4 (b), confirms the absence of competing states formed above the superconducting transition temperature $T_{\rm c}$.

V. ANALYSIS OF $\lambda^{-2}(T)$ DEPENDENCIES

The individual temperature dependencies of the inverse squared magnetic penetration depth λ^{-2} of $Mo_5Si_{3-x}P_x$, as obtained in TF-μSR experiments, are presented in Fig. 5. The parameters obtained from the fit of the $s-$wave gap model (Eq. 3 in the main text) to $\lambda^{-2}(T)$ data, namely the superconducting transition temperature $T_{\rm c}$, the zero temperature value of the inverse squared magnetic penetration depth $\lambda^{-2}(0)$, and the superconducting energy gap $\Delta(0)$, are shown at the corresponding panels.

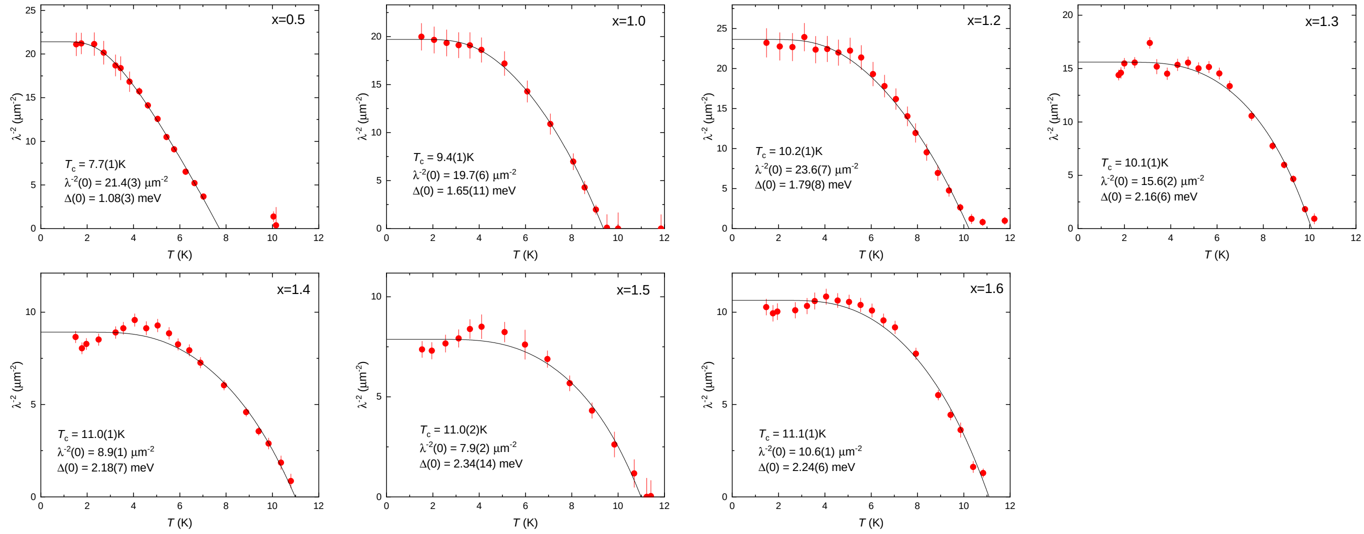

FIG. 5: Temperature dependencies of the inverse squared magnetic penetration depth λ^{-2} of $\mathrm{Mo_5Si_{3-\mathit{x}}P_{\mathit{x}}}$ as obtained in TF-μSR experiments. The solid lines are fits of Eq. 3 from the main text to $\lambda^{-2}(T)$ data. The fit parameters, namely the superconducting transition temperature T_c, the zero temperature value of the inverse squared magnetic penetration depth $\lambda^{-2}(0)$, and the superconducting energy gap $\Delta(0)$, are presented at each corresponding panel.

VI. ZERO-FIELD μSR MEASUREMENTS

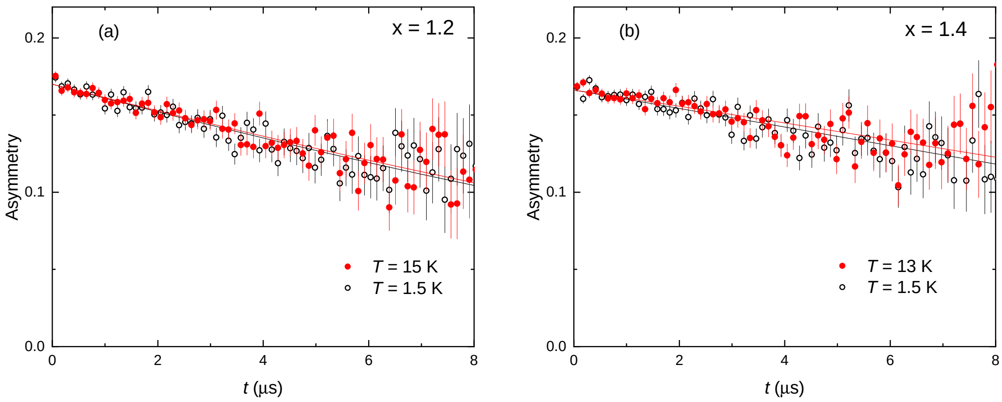

FIG. 6: Representative ZF-μSR time-spectra in the normal and the superconducting state of $x = 1.2$ and $x = 1.4$ $\mathrm{Mo_5Si_{3-\mathit{x}}P_{\mathit{x}}}$ samples. The solid lines are fits of Eq. 5 to the data.

The zero-field μSR measurements were performed in order to reveal a possible breaking of the time-reversal symmetry, which may indicate an unconventional superconducting state of $\mathrm{Mo_5Si_{3-\mathit{x}}P_{\mathit{x}}}$ system. The ZF-μSR time spectra for $x = 1.2$ and $x = 1.4$ samples, collected above the superconducting transition temperature T_c and at $T \simeq 1.5$ K, are presented in Fig. 6. Neither coherent oscillations nor fast decays could be seen, thus excluding any type of magnetic order or fluctuations.

The zero-field μSR spectra were fitted using the Gaussian Kubo-Toyabe (GKT) relaxation function,[3,4] describing the nuclear moment response, multiplied by an additional exponential term:

$$A(t) = A_0 \left[\frac{1}{3} + \frac{2}{3}(1 - \sigma_{\rm GKT}^2 t^2)\; e^{-\sigma_{\rm GKT}^2 t^2/2} \right] \; e^{-\Lambda t}. \tag{5}$$

Here, A_0 is the initial asymmetry, $\sigma_{\rm GKT}$ is the GKT relaxation rate, and Λ is the exponential relaxation rate. In the above equation, $\sigma_{\rm GKT}$ accounts for the nuclear moment contribution, which is assumed to be static within the μSR time window and independent on temperature.

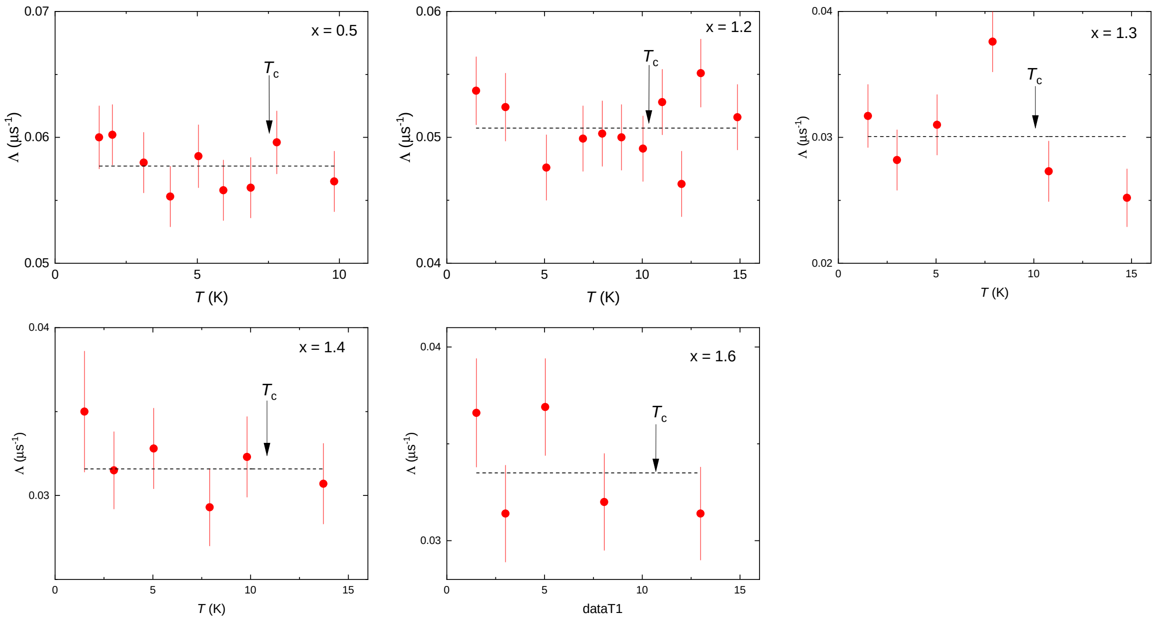

FIG. 7: Temperature dependencies of the exponential relaxation rate Λ of $Mo_5Si_{3-x}P_x$ as obtained in ZF-μSR experiments.

Figure 7 shows the temperature dependence of the exponential relaxation rate Λ of $Mo_5Si_{3-x}P_x$ samples. The absence of an additional μSR relaxation below T_c excludes a possible time-reversal symmetry breaking in the superconducting state of $Mo_5Si_{3-x}P_x$.

* Electronic address: rustem.khasanov@psi.ch
† Electronic address: bbruan@mail.ustc.edu.cn

[1] Bin-Bin Ruan, Jun-Nan Sun, Yin Chen, Qing-Song Yang, Kang Zhao, Meng-Hu Zhou, Ya-Dong Gu, Ming-Wei Ma, Gen-Fu Chen, Lei Shan, Zhi-An Ren, *Strong-coupling superconductivity with* $T_c \sim 10.8$ *K induced by P doping in the topological semimetal* Mo_5Si_3. Sci. China Mater. **65**, 3125 (2022).
`https://doi.org/10.1007/s40843-022-2102-8`

[2] W. L. McMillan, *Transition Temperature of Strong-Coupled Superconductors*, Phys. Rev. **167**, 331 (1968).
`https://doi.org/10.1103/PhysRev.167.331`

[3] A. Suter and B. M. Wojek, *Musrfit: A Free Platform-Independent Framework for* μ*SR Data Analysis*, Phys. Procedia **30**, 69 (2012).
`https://doi.org/10.1016/j.phpro.2012.04.042`

[4] R. S. Hayano, Y. J. Uemura, J. Imazato, N. Nishida, T. Yamazaki, and R. Kubo. *Zero-and low-field spin relaxation studied by positive muons.* Phys. Rev. B **20**, 850 (1979).
`https://doi.org/doi:10.1103/PhysRevB.20.850`
